# The Role of Tai Chi in Community Health Management: An Empirical Study on Residents’ Health Behaviors

**DOI:** 10.1093/geroni/igaf122.1829

**Published:** 2025-12-31

**Authors:** Ning Kang, Gong Chen, Dongmin Wang

**Affiliations:** Peking University, Beijing, Beijing, China (People’s Republic); Peking University, Beijing, Beijing, China (People’s Republic); Peking University, Beijing, Beijing, China (People’s Republic)

## Abstract

Findings indicate that Tai Chi plays a multifaceted role in community health management. First, it enhances residents’ health awareness, encouraging them to be more attentive to their well-being and adopt proactive health management strategies. Second, long-term Tai Chi practice fosters the development of healthy behaviors, including the establishment of regular exercise routines and positive lifestyle changes in diet and sleep habits. Additionally, Tai Chi demonstrates significant psychological benefits, effectively alleviating anxiety and stress while enhancing psychological resilience. As a group-based activity, Tai Chi also fosters social interactions within the community, strengthening neighborhood ties and contributing to the formation of a supportive health environment. This, in turn, facilitates a mutual-assistance model of community health management. Furthermore, some communities have integrated Tai Chi into their health management frameworks, combining it with health consultations and chronic disease management services, thereby expanding its application in health promotion. Consequently, Tai Chi serves not only as a form of physical exercise but also as a valuable tool for community health management, improving residents’ overall health at multiple levels.

**Conclusion:**

Tai Chi is more than just an exercise; it is a strategic tool for health management that fosters healthy behaviors, enhances residents’ physical and mental well-being, and strengthens social capital within communities. It is recommended that community health administrators leverage the advantages of Tai Chi by incorporating it into health promotion and chronic disease management programs. Optimizing health management strategies through Tai Chi can further improve the overall health of community residents.

